# Supplementary material for: Strong absorption and ultrafast localisation in NaBiS2 nanocrystals with slow charge-carrier recombination
Source: Nat Commun. 2022 Aug 24;13:4960. doi: 10.1038/s41467-022-32669-3 (PMC9402705; doi:10.1038/s41467-022-32669-3)
Supplement: Supplementary file 1 — Supplementary Information [file 41467_2022_32669_MOESM1_ESM.pdf]

Supplementary Information

Strong Absorption and Ultrafast Localisation in $NaBiS_2$ Nanocrystals with Slow Charge-Carrier Recombination

Yi-Teng Huang,[1,†] Seán R. Kavanagh,[2,3,4,†] Marcello Righetto,[5] Marin Rusu,[6] Igal Levine,[7] Thomas Unold,[6] Szymon J. Zelewski,[1,8] Alexander J. Sneyd,[1] Kaiwen Zhang,[9] Linjie Dai,[1] Andrew J. Britton,[10] Junzhi Ye,[1] Jaakko Julin,[11] Mari P. Napari,[12] Zhilong Zhang,[1] James Xiao,[1] Mikko Laitinen,[13] Laura Torrente-Murciano,[9] Samuel D. Stranks,[1,9] Akshay Rao,[1] Laura M. Herz,[5,13] David O. Scanlon,[2,4] Aron Walsh,[3,4] and Robert L. Z. Hoye[3,*]

[1.] Cavendish Laboratory, University of Cambridge, JJ Thomson Ave, Cambridge CB3 0HE, United Kingdom

[2.] Department of Chemistry, University College London, 20 Gordon Street, London WC1H 0AJ, United Kingdom

[3.] Department of Materials, Imperial College London, Exhibition Road, London SW7 2AZ, United Kingdom

[4.] Thomas Young Centre, University College London, Gower Street, London WC1E 6BT, United Kingdom

[5.] Department of Physics, University of Oxford, Clarendon Laboratory, Parks Road, Oxford, OX1 3PU, United Kingdom

[6.] Struktur und Dynamik von Energiematerialien, Helmholtz-Zentrum Berlin für Materialien und Energie, 14109 Berlin, Germany

[7]. Helmholtz-Zentrum Berlin für Materialien und Energie GmbH, Kekuléstraße 5, 12489 Berlin, Germany

[8.] Department of Semiconductor Materials Engineering, Faculty of Fundamental Problems of Technology, Wrocław University of Science and Technology, Wybrzeże Wyspiańskiego 27, 50-370 Wrocław, Poland

[9.] Department of Chemical Engineering and Biotechnology, University of Cambridge, Philippa Fawcett Drive, CB3 0AS, Cambridge, United Kingdom.

[10.] School of Chemical and Process Engineering, University of Leeds, LS2 9JT

[11.] Department of Physics, University of Jyväskylä, P.O. Box 35, 40014 University of Jyväskylä, Finland

[12.] School of Electronics and Computer Science, University of Southampton, Southampton SO17 1BJ, United Kingdom

[13.] Institute for Advanced Study, Technical University of Munich, Lichtenbergstrasse 2a, D-85748 Garching, Germany

[†]These authors contributed equally to this work.

* Email: r.hoye@imperial.ac.uk

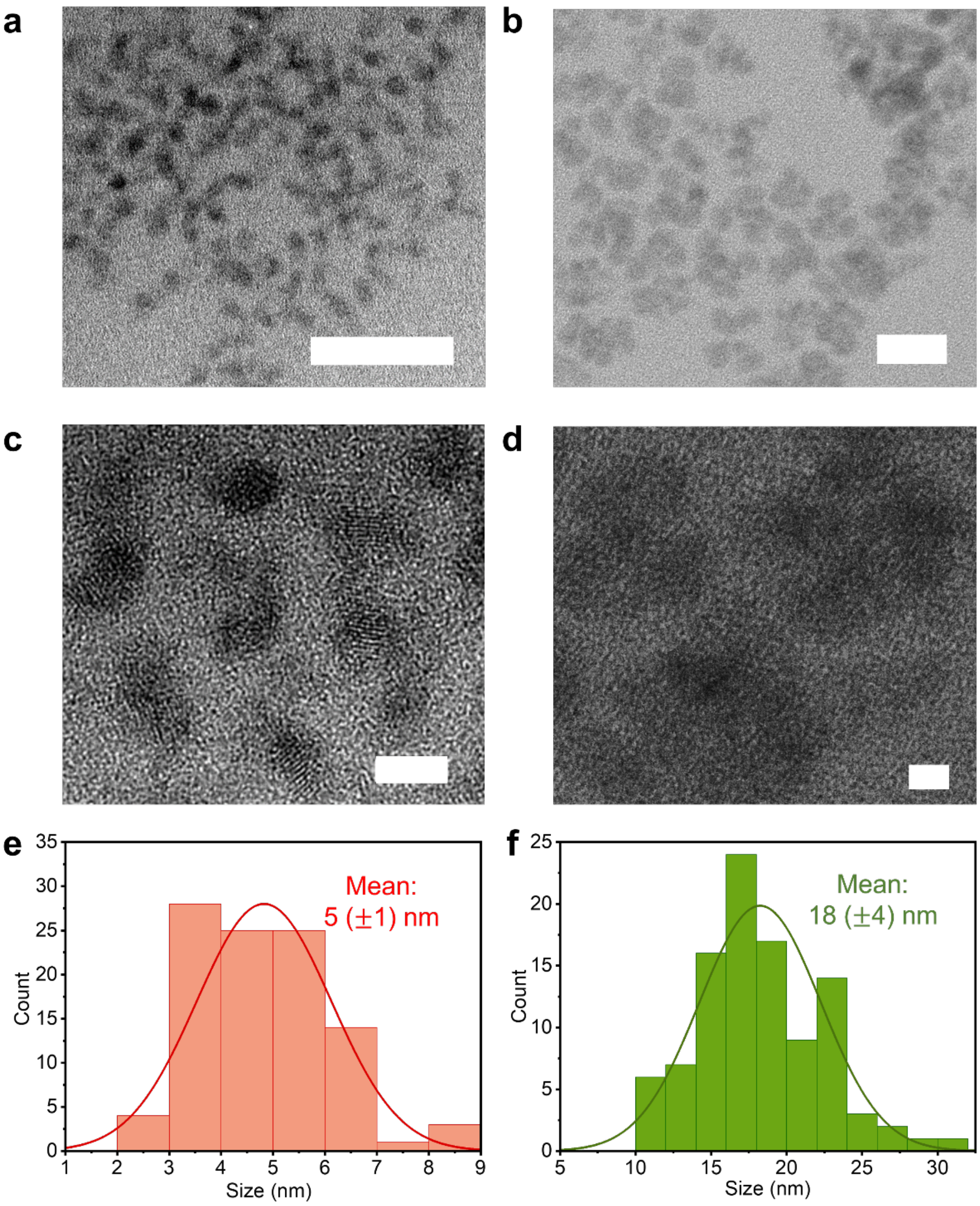

Supplementary Figure 1. Transmission electron microscopy (TEM) images and size distribution of $NaBiS_2$ NCs synthesised at different temperatures. **a**, **c** TEM images at different magnifications for $NaBiS_2$ NCs synthesised at 80 °C, and **e** the corresponding size distribution. **b**, **d** TEM images at different magnifications for $NaBiS_2$ NCs synthesised at 150 °C, and **e** the corresponding size distribution. The scale bar is 30 nm in **a** and **b**, and 5 nm in **c** and **d**, respectively.

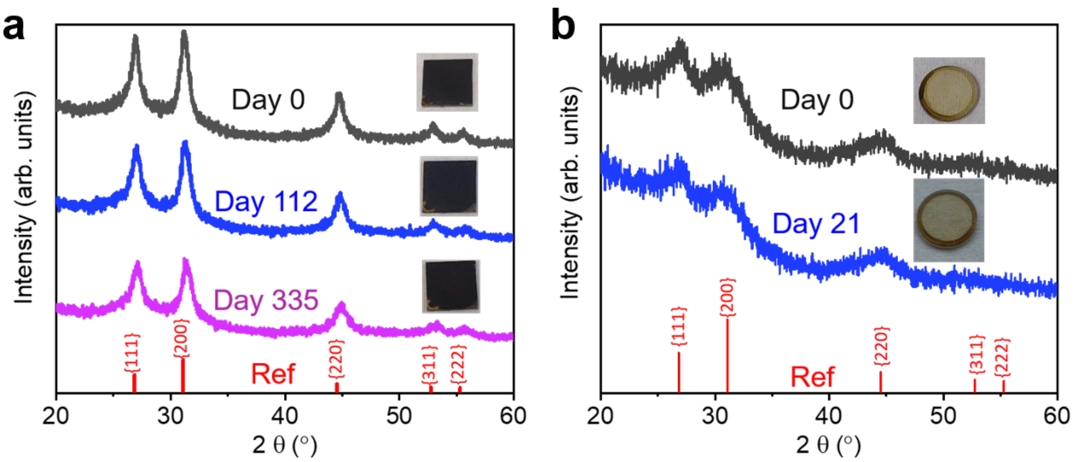

Supplementary Figure 2. Phase stability of $NaBiS_2$ NC films. X-ray diffraction (XRD) patterns and photographs of $NaBiS_2$ NC films synthesised at **a** 150 °C and **b** 80 °C. Samples stored in air for up to 335 days, where the relative humidity varied between 60 and 70% (Day 0 represents the results for the films on the same day of preparation). Reference pattern obtained from Ref. 1.

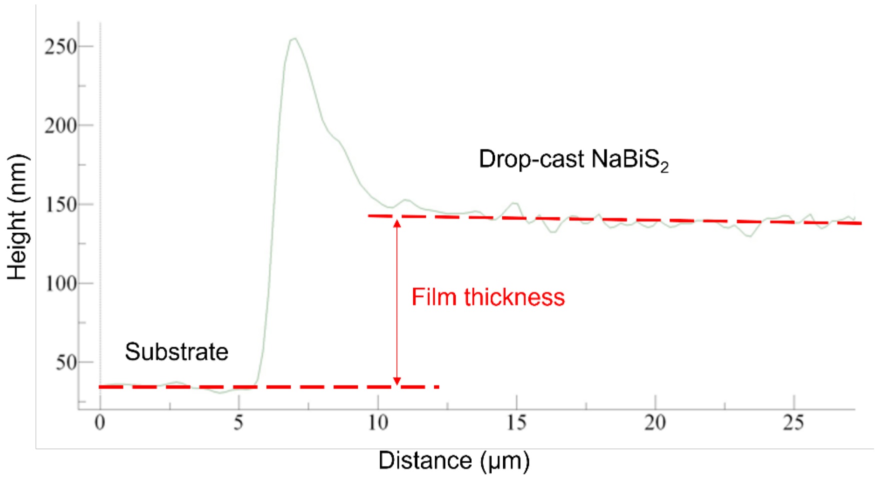

Supplementary Figure 3. Profile of the step-edge from the substrate to drop-cast $NaBiS_2$ film (dried in an Ar-filled glovebox) measured by atomic force microscopy (AFM). The film thickness is homogeneous with a thickness variation less than 10 nm. The peak at the step edge was from the accumulated NCs when using a blade to scratch the film in order to create a step-edge.

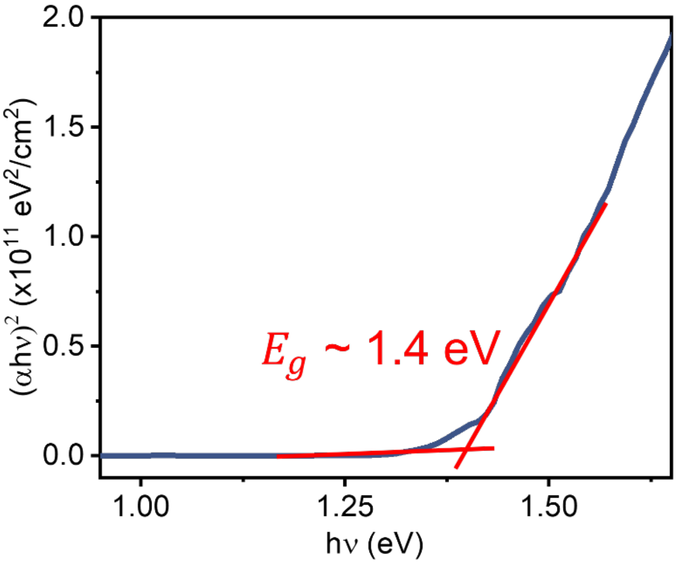

Supplementary Figure 4. Tauc plot of the $NaBiS_2$ NC film based on a direct-allowed transition.

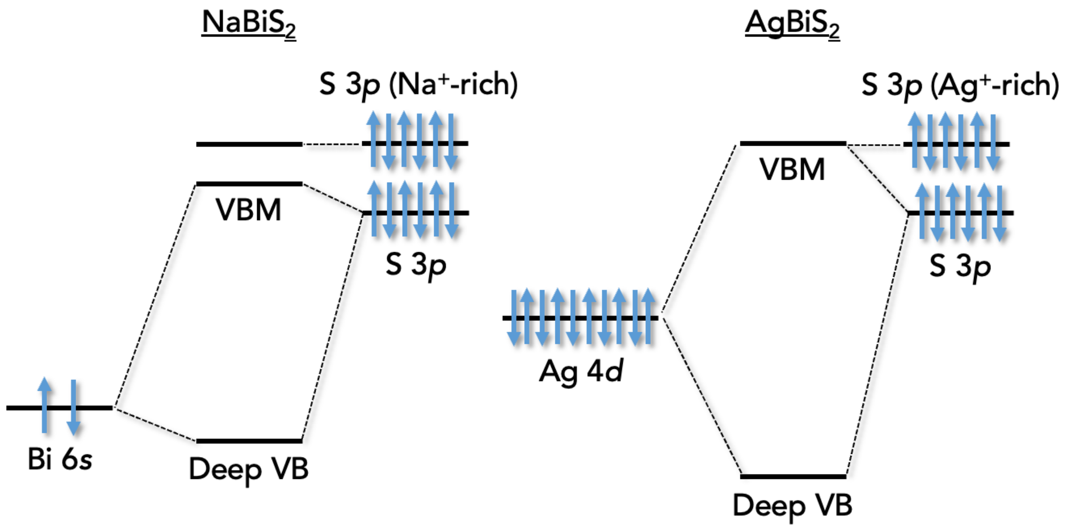

Supplementary Figure 5. Schematic Molecular Orbital (MO) diagram of the upper valence band (VB) electronic structure in $NaBiS_2$ **(left)** and $AgBiS_2$ **(right)**. Sulphur orbitals with high coordination of A^+ cations (denoted as "S 3*p* A^+-rich") have higher energy owing to reduced Coulombic potential from the lower-charge cations (compared to Bi^{3+}). From this diagram, it is shown that due to the greater Ag 4*d* – S 3*p* repulsion,

$AgBiS_2$ has a larger bandwidth in the VB, such that S 3*p* (Ag^+-rich) remains within the VB, whereas in $NaBiS_2$ S 3*p* (Na^+-rich) forms a localised state above the VB maximum.

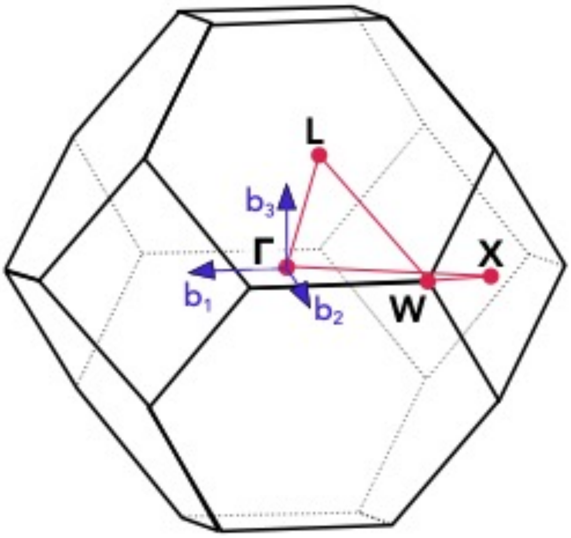

Supplementary Figure 6. First Brillouin Zone (BZ) of an F-centred cubic lattice, to which the disordered $NaBiS_2$ crystal space group ($Fm\overline{3}m$) belongs. The high-symmetry BZ points and band path included in the electronic band structure (Fig. 2a) are shown in colour. Vectors $b_{1,2,3}$ denote the cell vectors of the Wigner-Seitz primitive cell.

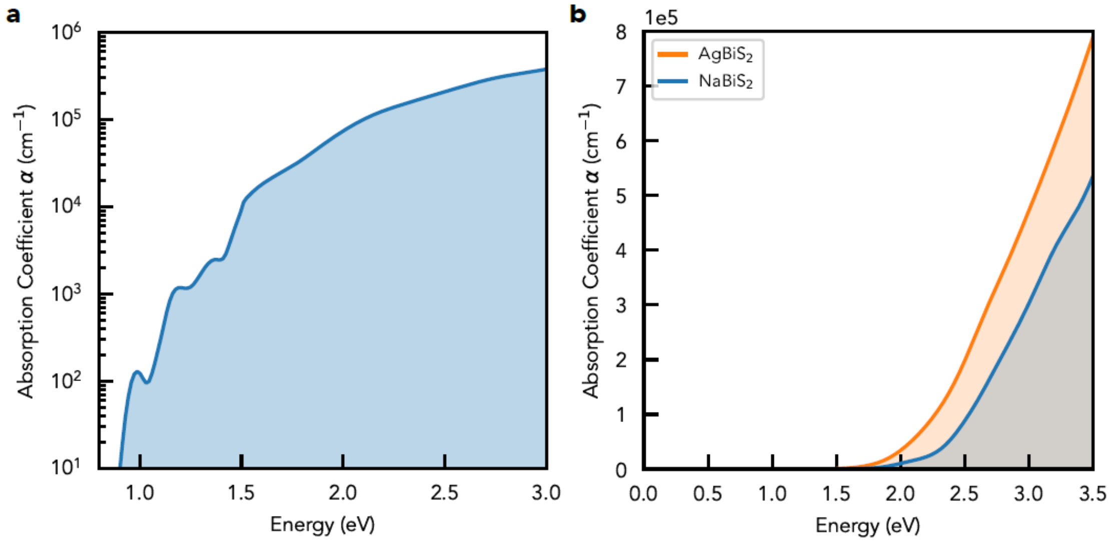

Supplementary Figure 7. Optical absorption profiles. **a** Calculated absorption coefficient spectrum of disordered $Fm\overline{3}m$ $NaBiS_2$. **b** Comparison of the calculated absorption onset for $AgBiS_2$ and $NaBiS_2$ for the same random-disorder cation

arrangement. We note that the $NaBiS_2$ produced in this work shows stronger absorption onsets than previously-reported for $AgBiS_2$ in Ref. 2. However, it was shown[2] that the absorption is highly sensitive to cation arrangements in these materials. Thus, the differing disorder energetics, cation distributions and/or size effects in the synthesised samples likely explain the difference between theory and experiment in terms of the comparison between the absorption coefficient of $NaBiS_2$ *vs.* $AgBiS_2$.

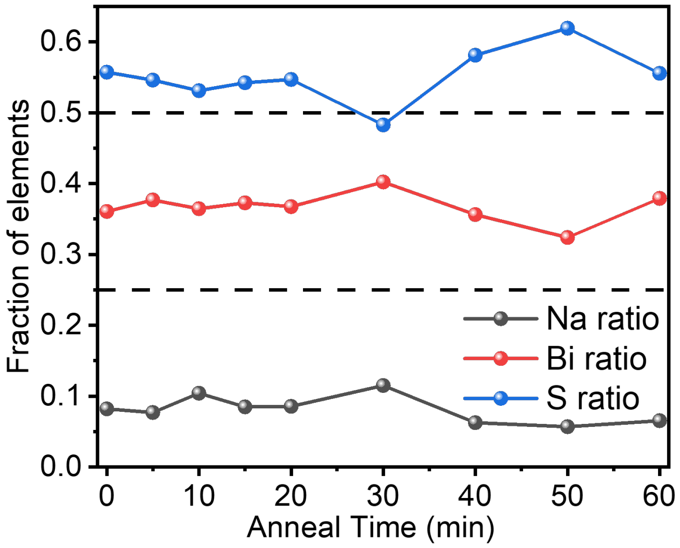

Supplementary Figure 8. Fraction of Na, Bi and S elements (the sum of the fractions of these elements has been normalised), measured from Time-of-Flight Elastic Recoil Detector Analysis (ToF-ERDA) of $NaBiS_2$ NC films annealed at 100 °C in an Ar-filled glovebox for different times. The two dashed lines represent the fractions (0.25 and 0.5) corresponding to the ideal values for stoichiometric Na/Bi and S, respectively, The ToF-ERDA measurements were taken using a 11.9 MeV $^{63}Cu^{6+}$ beam in mirror geometry (20° between incident beam and sample surface), with further details in Ref. 3. The measurements were analysed using the Potku analysis software[4]. From these measurements, it can be seen that the bulk composition was deficient in Na. We note that in our measurements of $NaBiS_2$ NC films under ultra-high vacuum by conventional

XPS or energy-dispersive X-ray spectroscopy (EDX), the films were also found to be Na-deficient, which contrasts to the Na-rich surface in ambient-pressure XPS measurements. In addition, a significantly reduced Na peak intensity in the XPS spectrum could be seen in the sample stored in an ultra-high vacuum chamber overnight compared to the one stored in ambient air. These results suggest that Na is easily removed under ultra-high vacuum. In the ToF-ERDA measurements, samples were kept under high vacuum (approx. 10^{-8} mbar), and beam damage on samples was also observed. Therefore, the fraction of elements here may not be very accurate owing the removal of Na or other elements. Nevertheless, these measurements showed that Na, Bi and S were all present in the bulk of $NaBiS_2$ NC films and there was no significant trend in any changes in composition after annealing. It should also be noted that the majority of the elements present in these measurements were H and C originating from the long-chain ligands present on the NCs, while Na, Bi, and S only occupied a small fraction of total elements detected.

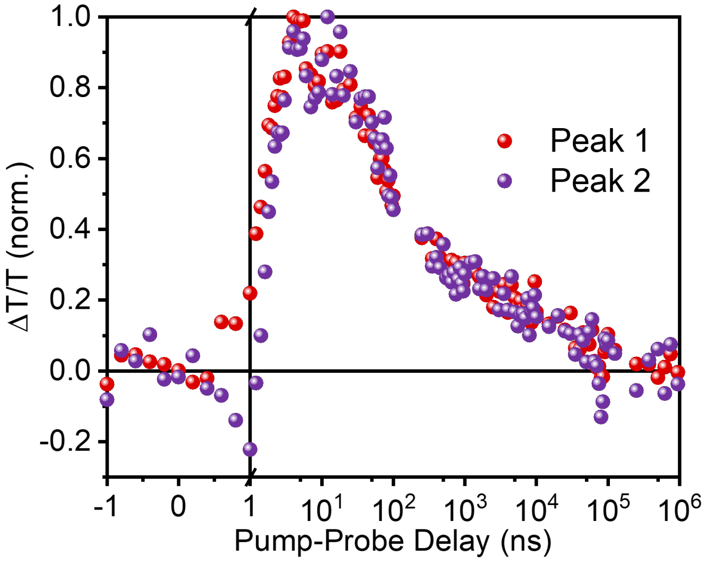

Supplementary Figure 9. Normalised signal kinetics extracted from the two ground state bleach (GSB) peaks (peak 1 and peak 2) in long-time transient absorption

spectrum of $NaBiS_2$ NC films. The signals for peak 1 and peak 2 were acquired by averaging over the wavelength ranges of 800–860 nm and 860–960 nm, respectively. Each signal kinetics was normalised to its maximum $\Delta T/T$ value.

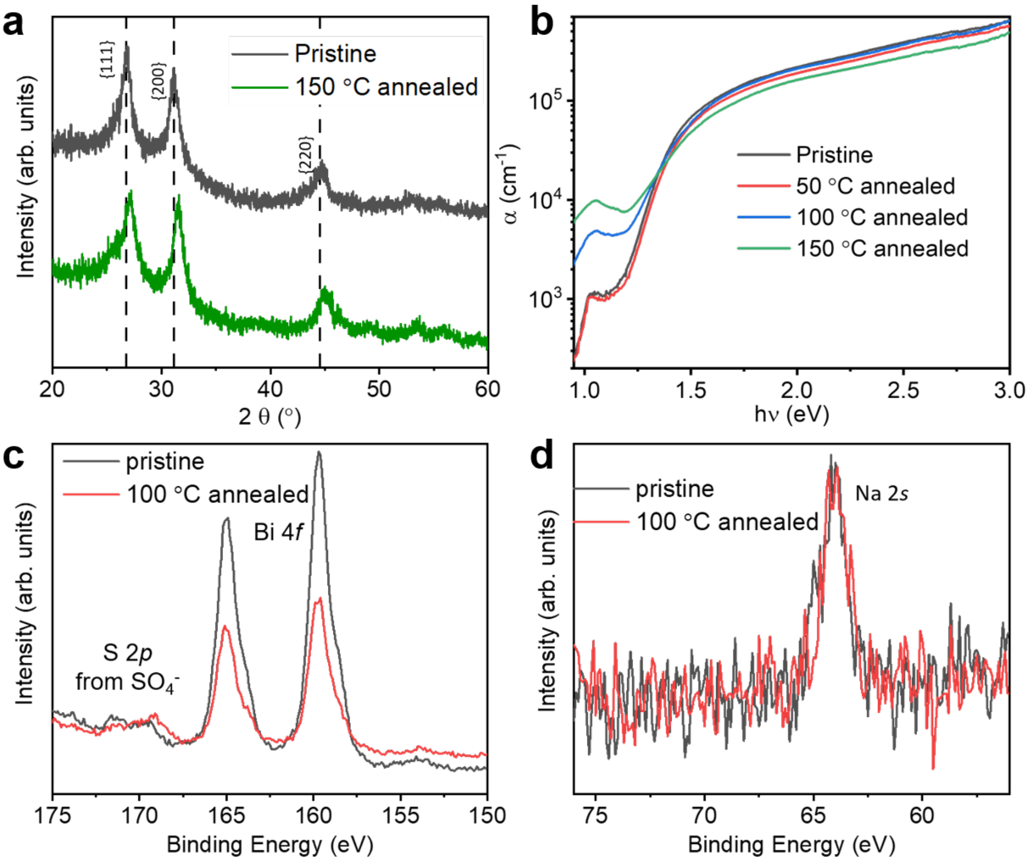

Supplementary Figure 10. Effect of post-annealing treatment (for 1 hour in an Ar-filled glovebox) on $NaBiS_2$ NC films. **a** XRD patterns of pristine and post-annealed (at 150 °C) $NaBiS_2$ NC films. The peak positions for the pristine $NaBiS_2$ film are displayed as dash-lines for comparison. **b** Absorption coefficient α of pristine and annealed $NaBiS_2$ films at different temperatures. **c** Ambient-pressure X-ray photoemission spectroscopy (XPS) measurements, showing the Bi 4*f* and S 2*p* core levels, as well as **d** the Na 2*s* peak of pristine and 100 °C annealed $NaBiS_2$ films.

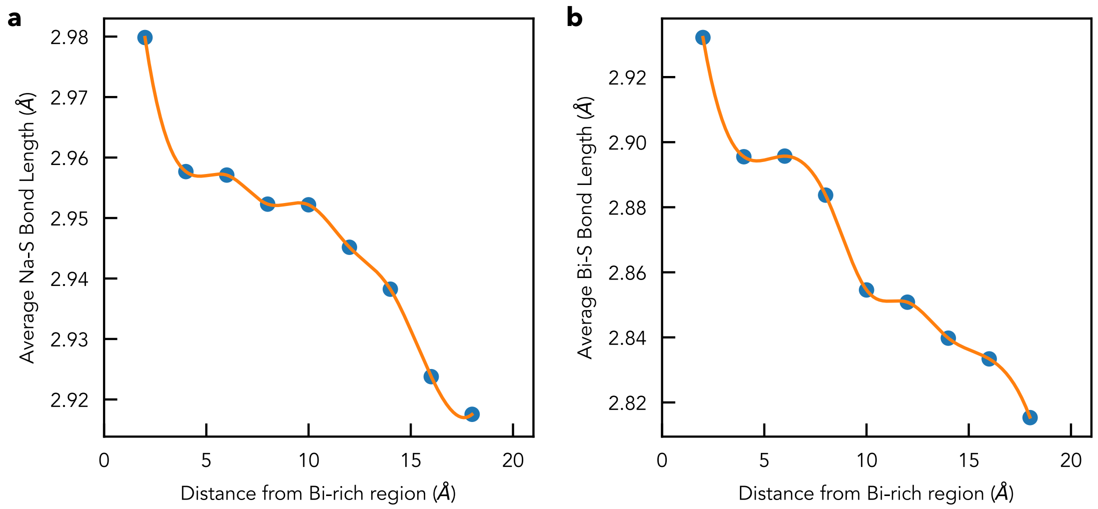

Supplementary Figure 11. Average calculated **a** Na–S and **b** Bi–S bond lengths for disordered ($Fm\bar{3}m$) $NaBiS_2$ in a representative 400-atom SQS supercell relaxed using hybrid DFT, as a function of distance from the Bi-rich region of the supercell. In agreement with observations for $AgBiS_2$ [2], regions of greater cation homogeneity (far from the Bi-rich region) show decreased cation-anion bond lengths.

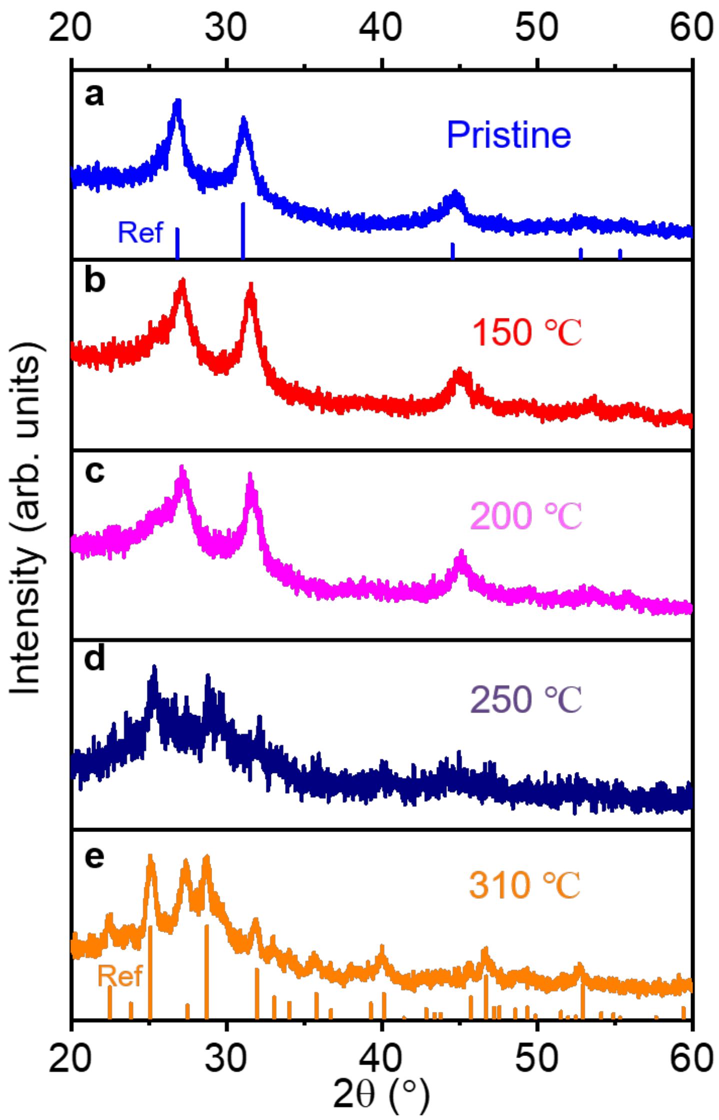

Supplementary Figure 12. XRD patterns of $NaBiS_2$ films post-annealed at temperatures from **a** 150 °C up to **e** 310 °C for 1 hour in an Ar-filled glovebox. The rocksalt cubic phase of $NaBiS_2$ ($Fm\bar{3}m$) starts to transform into orthorhombic Bi_2S_3 (*Pbnm*) after annealing at 250 °C or higher. The reference pattern for disordered rocksalt $NaBiS_2$ (ICSD data base, Coll. Code: 616841)[1] and Bi_2S_3 (ICSD data base, Coll. Code: 89324)[5] are displayed in **a** and **e**, respectively.

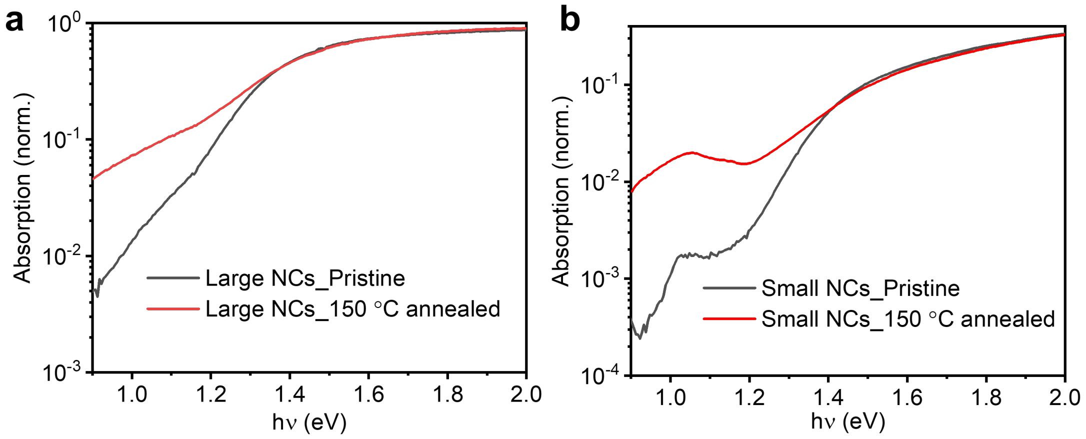

Supplementary Figure 13. Effect of post-annealing on sub-bandgap absorption in large *vs.* small $NaBiS_2$ NCs. Photothermal deflection spectroscopy (PDS) absorbance spectrum of $NaBiS_2$ films composed of **a** large NCs (mean size ~18 nm) and **b** small NCs (mean size ~5 nm). These spectra are normalised to the absorbance at 3.1 eV. The spectrum of the pristine film and film annealed at 150 °C for 1 hour in an argon-filled glovebox are displayed in each sub-Figure.

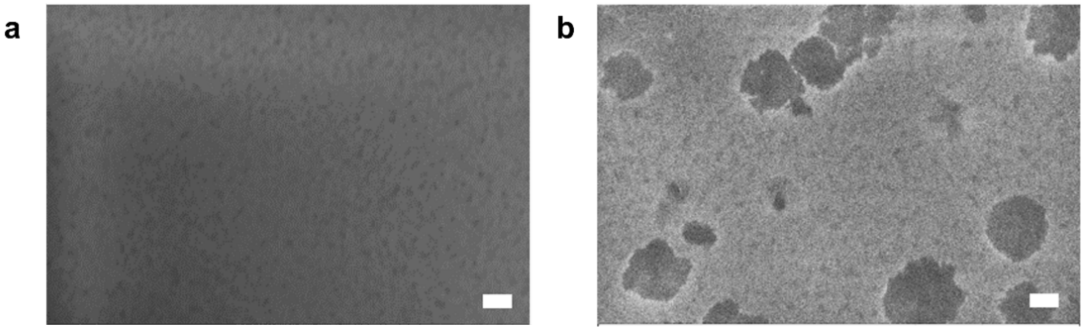

Supplementary Figure 14. Scanning electron microscopy images of **a** pristine and **b** 100 °C annealed $NaBiS_2$ NC films. Scale bar is 2 μm in both cases.

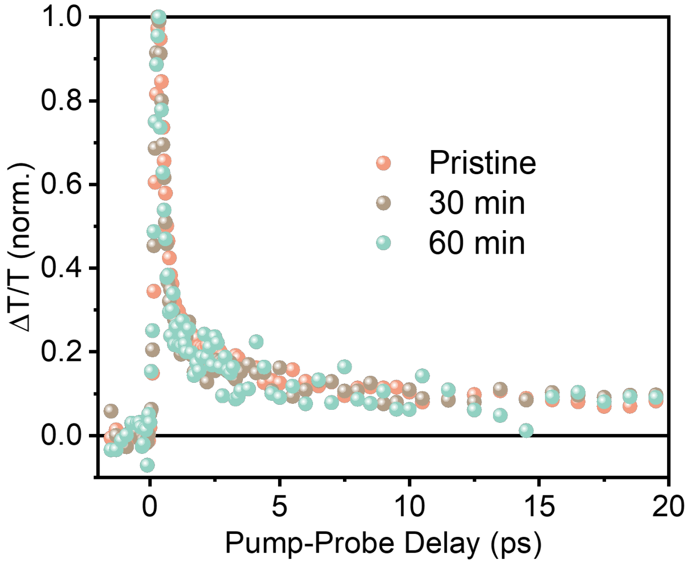

Supplementary Figure 15. Normalised photo-induced absorption (PIA) kinetics extracted from short-time transient absorption measurements of pristine and annealed $NaBiS_2$ NC films at 100 °C in an Ar-filled glovebox for different times (30 and 60 min). All the samples were excited by a 400 nm wavelength pump at a fluence of 12 μJ cm^{-2} $pulse^{-1}$ and all the kinetics are normalised to their minimum values.

Supplementary Note 1. SLME calculations

The calculations are based on the method reported in Ref. 6. In brief, at the radiative limit, the short-circuit current density $J_{\mathrm{SC\_r}}$ and $V_{\mathrm{OC\_r}}$ can be calculated based on the equations below:

$$J_{\mathrm{SC\_r}} = q \int a(E) I_{\mathrm{AM1.5G}}(E)\, dE \tag{S1}$$

$$V_{\mathrm{OC\_r}} = \frac{kT}{q} ln\left(\frac{J_{\mathrm{SC\_r}}}{J_0} + 1\right) \tag{S2}$$

where $a(E)$ is the absorptance of the material, $I_{\mathrm{AM1.5G}}$ is the photon flux density of AM 1.5G solar spectrum, q, k, and T are the elementary charge, Boltzmann constant, and temperature, respectively. J_0 is the reverse saturation current and can be expressed by Equation S3:

$$J_0 = q \int a(E) \Phi_{\mathrm{BB}}(E) dE \tag{S3}$$

with $\Phi_{\mathrm{BB}}(E)$ the spectral photon flux of blackbody radiation at 300 K, which is

$$\Phi_{\mathrm{BB}}(E) = \frac{1}{4\pi^2 \hbar^3 c^2} \frac{E^2}{e^{\frac{E}{kT}} - 1} \tag{S4}$$

with $\hbar$ and c the reduced Planck constant and speed of light. In the SLME model, we assume $a(E) = 0$ for all E below the bandgap E_{g} of a material, and the reverse saturation current J_0 will be modified into $J_0 e^{\frac{q\Delta}{kT}}$ for all indirect-bandgap materials with a difference Δ between their fundamental bandgaps and direct transition energy (to approximate non-radiative losses). The SLME can then be calculated based on $P_{\mathrm{m}}/P_{\mathrm{in}}$ with P_{m} and P_{in} the maximum output power density and one-sun power density. Here, $P_{\mathrm{m}} = J_{\mathrm{m}} V_{\mathrm{m}}$, where J_{m} and V_{m} are the current density and voltage at the maximum power point, which can associate with $J_{\mathrm{SC\_r}}$ and $V_{\mathrm{OC\_r}}$ by the following equations:

$$J_{\mathrm{m}} = J_{\mathrm{SC_r}} \left(\frac{V_{\mathrm{OC_r}} - \log(V_{\mathrm{OC_r}} + 1)}{V_{\mathrm{OC_r}} - \log(V_{\mathrm{OC_r}} + 1) + 1} \right) \tag{S5}$$

$$V_{\mathrm{m}} = \frac{kT}{q} \left(V_{\mathrm{OC_r}} - \log\left(V_{\mathrm{OC_r}} - \log\left(V_{\mathrm{OC_r}} + 1 \right) + 1 \right) \right) \tag{S6}$$

Note that all the SLMEs in this work were calculated based on the measured absorption coefficient spectra/bandgaps of the PV absorbers either from our own experiments or from literature reports for a single film, rather than complete devices based on the transfer matrix formalism[2], which might give higher values owing to light trapping or interference effects within/between each device layer.

Supplementary Table 1. SLME of various PV absorbers at 30 nm thickness without and with consideration of the non-radiative loss from the difference between the direct transition and indirect bandgap (Δ). Only absorbers with indirect bandgaps need to have this effect accounted for. As detailed in Supplementary Note 1, Δ could increase

the reverse saturation current J_0 and lower the SLMEs. We note again that all the SLMEs here are not calculated for complete devices based on the transfer matrix formalism.

Absorber	w/o loss from Δ	w/ loss from Δ
$NaBiS_2$	26.17%	25.96% (Δ = 0.01 eV)
$AgBiS_2$	21.05%	18.05% (Δ = 0.11 eV)
CdTe	11.13%	---
Sb_2Se_3	6.94%	---
CZTSe	6.76%	---
InP	9.42%	---
PbS	9.11%	---
GaAs	9.51%	---
CIGS	11.91%	---
MAPI	12.39%	---

Supplementary Note 2. Elliott Model Fitting

Even though the Tauc method has been widely used to determine the bandgaps in perovskite-inspired materials[7–9], recent work has indicated that the Tauc method tends to systematically underestimate the bandgaps of materials[10,11] because it applies the linear fit to the logarithmic absorption curve and does not take into account of the excitonic contributions[11]. To account for the contributions from excitonic states, we applied the Elliott model and fitted the onset of the absorption coefficient spectrum reported in Fig. 1c (main text). Based on the Elliott model[12], the absorption onset $\alpha(E)$ was contributed from both excitons $\alpha_x(E)$ and continuum states $\alpha_c(E)$ so that

$$\alpha(E) = \alpha_x(E) + \alpha_c(E) \quad \text{(S7)}$$

Following the treatment described by Wright et al.[11], each of these terms can be described as a function of the exciton binding energy E_b and the bandgap of the material E_g by

$$\alpha_x(E, E_\mathrm{g}, E_\mathrm{b}) = \frac{\alpha_0}{E}\sum_{j=1}^{\infty}\frac{4\pi E_\mathrm{b}^{3/2}}{j^3}\delta\left(E - \left[E_\mathrm{g} - \frac{E_\mathrm{b}}{j^2}\right]\right) \tag{S8}$$

$$\alpha_c(E, E_\mathrm{g}, E_\mathrm{b}) = \frac{\alpha_0}{E}\xi(E)c_0^{-1}JDoS(E) \tag{S9}$$

where α_0 is a constant, and j is a positive integer. $JDoS(E)$ is the joint density of states defined as

$$JDoS(E) = \begin{cases} \frac{c_0}{1-b(E-E_\mathrm{g})}\sqrt{E - E_\mathrm{g}} & for\ E > E_\mathrm{g} \\ 0 & \text{otherwise} \end{cases} \tag{S10}$$

with c_0 determined by the reduced effective mass $\mu = \frac{1}{m_e} + \frac{1}{m_h}$ (m_e and m_h is the effective electron and hole mass as defined in the main text) by $c_0 = \frac{2}{(2\pi)^2}\left(\frac{2\mu}{\hbar^2}\right)^{3/2}$, and b represents the non-parabolicity coefficient as described in Ref. 13. Here, for Equation (S9), the limit $b \to 0$ recovers the conventional density of state for parabolic band dispersion. Following the theory developed by Elliott[12], the $JDoS(E)$ term in Equation (S8) is further modified by a Coulombic enhancement factor $\xi(E)$ to account for electron-hole Coulomb interactions

$$\xi(E) = \frac{2\pi\sqrt{\frac{E_b}{E-E_g}}}{1-exp\left(-2\pi\sqrt{\frac{E_b}{E-E_g}}\right)} \tag{S11}$$

Furthermore, absorption broadening due to various effects can be considered by convoluting $\alpha(E)$ with a Gaussian distribution of width Γ. A more detailed discussion of the resulting Elliott fitting equation can be found in Ref. 11,14. The final convoluted function is then fitted to the absorption onset by adopting the least-squares minimisation method.

The Elliott fit to our experimental absorption data is shown in the red dashed line in Supplementary Fig. 16 below with the obtained parameters E_g = 1.57 eV, E_b = 0.012 eV, Γ = 0.13 eV, b = 0.35 eV^{-1}. The excitonic and continuum contributions are also respectively represented by the violet and purple shaded areas. As mentioned earlier, the fitted E_g (1.57 eV) is around 170 meV higher than that estimated from the Tauc plot (1.4 eV, Supplementary Fig. 4), and the fitted E_b (12 meV) is higher than the value reported for bulk $NaBiS_2$ (1-6 meV)[15] in the literature, but lower than the value we calculated (27 meV) using the Wannier-Mott hydrogenic model. Compared with the broadening parameters Γ reported in other perovskite-inspired materials (e.g., 75 meV for $Cs_2AgBiBr_6$, and 90 meV for Cu_2AgBiI_6[10,11]), disordered $NaBiS_2$ exhibits a high Γ of 130 meV, which can be due to cation disorder and/or disperse size distribution of the nanocrystals. Moreover, the obtained b value (0.35 eV^{-1}) is significantly different from zero, thus implying non-negligible deviations from parabolic band dispersion. Based on these results, we should note that some degree of inaccuracy must be introduced when fitting E_g and E_b values.

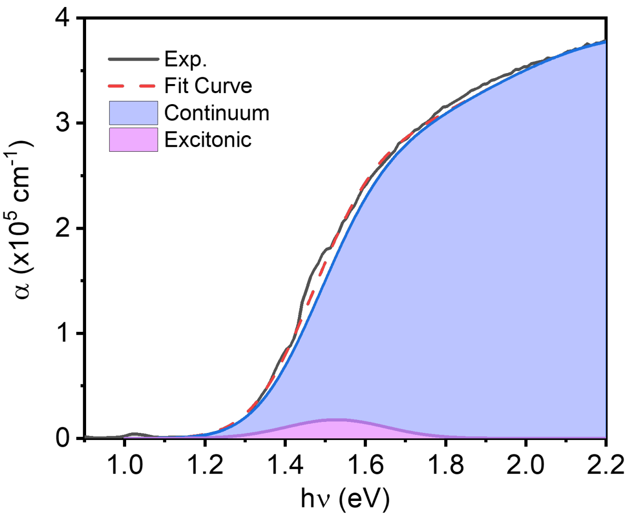

Supplementary Figure 16. Elliott fit to the absorption coefficient spectrum onset of $NaBiS_2$ NC thin film. The experimental data is plotted in dark grey (Exp.), and the

Elliott fit is plotted as the red dashed line. Excitonic and continuum contributions to the Elliott fit are plotted as the violet and purple shaded areas. The fitted parameters are E_g = 1.57 eV, E_b = 0.012 eV, Γ = 0.13 eV, b = 0.35 eV^{-1}.

Supplementary Note 3. Short-time transient absorption signal decomposition

To interpret the carrier kinetics more clearly, we applied singular-value decomposition (SVD) analysis on our short-time transient absorption (TA) results. Given a 2D TA spectrum as shown in Supplementary Fig. 17, SVD analysis can determine the number of principal components within a complex TA spectrum and extract their wavelength-dependent amplitudes as well as the associated kinetics[16]. Python function (numpy.linalg.svd) was used here to decompose the 2D TA spectrum into the product of 'wavelength component matrix', 'singular values matrix', and 'time evolution matrix'. As can be seen in Fig. 4a, two principal components A and B can be extracted from our 2D short-time TA spectrum (other components exhibit singular values that are too small and will evolve like noise). Component A has a negative amplitude and shows a very similar spectrum to the overall short-time TA spectrum in Fig. 3c in main text, while component B has a positive peak near 870 nm. We believe that component B may result from free carrier bleaching at the band edges in $NaBiS_2$ for three reasons: i) it has a single positive peak at the energy (870 nm, 1.43 eV), which is close to the 1.4 eV bandgap of $NaBiS_2$, ii) charge-carrier bleaching should occur at very early time (a few picoseconds after photoexcitation) rather than emerging after a few nanoseconds, as in the case of the ground state bleach (GSB) signals observed in the long-time TA measurements, and iii) component B shows a mono-exponential decay, suggesting a Shockley-Read-Hall recombination process, which is also expected in general GSB kinetics at low charge-carrier density.

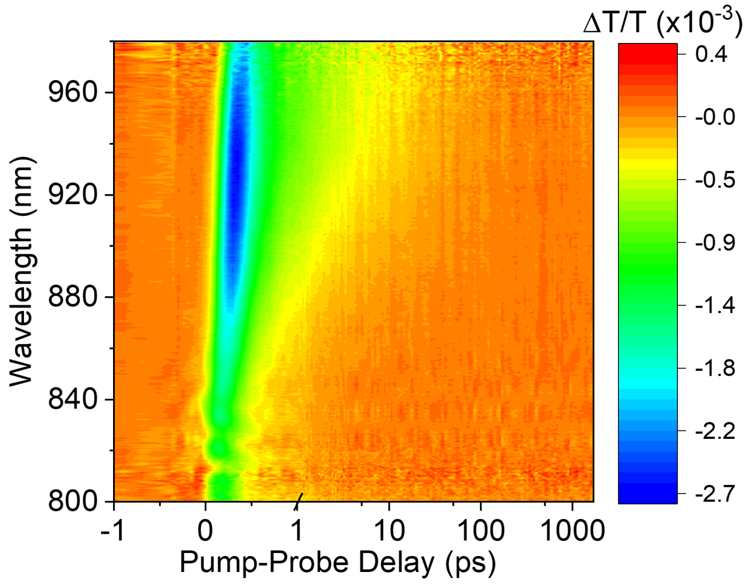

Supplementary Figure 17. A 2D short-time TA spectrum including both wavelength and kinetics information.

Supplementary Note 4. Charge-carrier mobility and the two-level mobility model

To determine the electron-hole sum mobility from optical pump terahertz probe (OPTP) measurements, we used the approach described by Wehrenfennig et al.[17]. Here, we converted the $\left(\frac{\Delta T}{T}\right)$ OPTP traces to photoconductivity traces $\Delta\sigma$. For thin films on a substrate (z-cut quartz, in our case) and measurements in vacuum, the approximated sheet photoconductivity can be expressed as

$$\Delta\sigma = -\frac{\epsilon_0 c(n_q+n_v)}{t_{\text{film}}}\left(\frac{\Delta T}{T}\right) \tag{S12}$$

where t_{film} is the thickness of the studied thin film, and $n_v = 1$, $n_q = 2.13$ are the refractive indices of z-cut quartz and vacuum, respectively[18]. The extracted photoconductivity is proportional to the electron-hole sum mobility μ and to the charge-carrier density n, as defined by the equation $\Delta\sigma = en\mu$. Hence, the photogenerated carrier density needs to be determined to access the electron-hole sum mobility. This is done by estimating the number of initial charge-carriers:

$$N = \varphi \frac{E\lambda}{hc}\left(1 - R_{\text{pump}} - T_{\text{pump}}\right) \tag{S13}$$

Here, φ is the photon-to-charge branching ratio (i.e., the ratio between photogenerated charges and absorbed photons), E is the excitation energy per pulse, and $\varepsilon = hc/\lambda$ is the energy of a photon with wavelength λ, and R_{pump} and T_{pump} are the reflectance and transmittance of the sample at 400 nm. This numeric density is converted to the initial charge-carrier density n_0 by dividing the film thickness t_{film} and the effective overlap area between THz and pump beam A_{eff}. Therefore, the resulting effective electron-hole sum mobility can be then expressed as:

$$\varphi\mu = -\epsilon_0 c\left(n_q + n_v\right)\frac{A_{\text{eff}}hc}{eE\lambda\left(1-R_{\text{pump}}-T_{\text{pump}}\right)}\left(\frac{\Delta T}{T}\right) \tag{S14}$$

To describe more in detail the observed ultrafast localisation process, we also applied the two-level mobility model developed by Wright, Buizza and co-workers[10]. In this model, the observed $\Delta\sigma$ signal is interpreted as the sum photoconductivity for a delocalized state and a localized state (Supplementary Fig. 18). Each one of them has a definite population and mobility: $(n_{\text{del}}, \mu_{\text{del}})$ and $(n_{\text{loc}}, \mu_{\text{loc}})$, respectively. Hence, the photoconductivity of the material can be described as $\Delta\sigma = e(n_{\text{del}}\mu_{\text{del}} + n_{\text{loc}}\mu_{\text{loc}})$. Considering a low excitation fluence regime, where the recombination from the localized state is predominantly monomolecular, the carrier population is described by the set of coupled rate equations:

$$\begin{cases} \frac{dn_{\text{del}}}{dt} = -k_{\text{loc}}n_{\text{del}}(t) \\ \frac{dn_{\text{loc}}}{dt} = k_{\text{loc}}n_{\text{del}}(t) - k_1 n_{\text{loc}}(t) \end{cases} \tag{S15}$$

Here, k_{loc} and k_1 are the localisation and monomolecular recombination rates, respectively. We note that, since $NaBiS_2$ does not show any detectable photoluminescence, k_1 can be assigned to non-radiative recombination of the localised charge carriers, possibly *via* a thermally-assisted process. As described in

detail in Refs 10,11, by combining Equation S14 with the solution of Equation S15, the resulting $\Delta T/T$ signal can be described as:

$$\frac{\Delta T}{T} = -\frac{e n_0 t_{\mathrm{film}}}{\epsilon_0 c(n_q+n_v)}\left(\left(\mu_{\mathrm{del}} - \frac{\mu_{\mathrm{loc}}k_{\mathrm{loc}}}{k_{\mathrm{loc}}-k_1}\right)e^{-k_{loc}t} + \frac{\mu_{\mathrm{loc}}k_{\mathrm{loc}}}{k_{\mathrm{loc}}-k_1}\, e^{-k_1 t}\right) \tag{S16}$$

Here, the values of $n_0 \times t_{\mathrm{film}}$ were obtained by applying Equation S13 and dividing the obtained value for the effective excitation area. For the fitting data reported in Fig. 3e of the main text, $n_0 \times t_{\mathrm{film}}$value was 1.5×10^{14}, 8.9×10^{13}, and 5.1×10^{13} cm^{-2} respectively for various fluences. For fit reported in Fig. 4e, the obtained value is $n_0 \times t_{\mathrm{film}} = 4.8 \times 10^{13}$ cm^{-2}. Finally, all the fits to the experimental data reported in the main text (Fig. 3e, 4e) were achieved by convoluting the decay function resulting from Equation S16 with a Gaussian broadening (250 fs) function to model the instrumental response function, as described in Ref. 10.

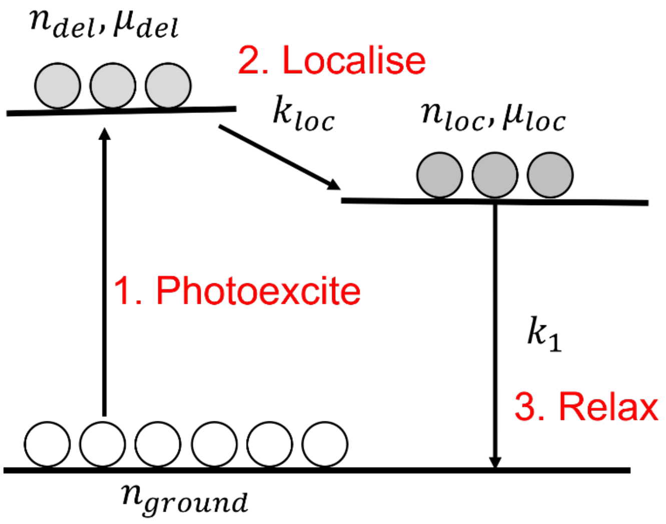

Supplementary Figure 18. Schematic of the two-level mobility model.

Supplementary Table 2. OPTP dynamics fitting parameters for spin-coated, pristine and annealed (30 min and 60 min) $NaBiS_2$ NC films. Data were fitted using the two-level mobility model described above in Supplementary Note 4.

Sample	μ_{del} ($cm^2V^{-1}s^{-1}$)	μ_{loc} ($cm^2V^{-1}s^{-1}$)	k_{loc} (ps^{-1})	$1/k_1$ (ps)
**Spin-coat**	0.29 ± 0.02	0.03 ± 0.02	2.5 ± 0.6	5 ± 2
**Pristine**	0.14 ± 0.01	0.03 ± 0.02	2.1 ± 0.7	4 ± 2
**Anneal 30 min**	0.25 ± 0.01	0.02 ± 0.02	2.7 ± 0.5	12 ± 3
**Anneal 60 min**	0.27 ± 0.01	0.03 ± 0.02	1.3 ± 0.2	15 ± 6

Supplementary Table 3. Extracted parameters and the estimated effective lifetime obtained by fitting a biexponential model to the normalised ground state bleach (GSB) kinetics of $NaBiS_2$ NC films annealed for different times. Measured by long-time transient absorption spectroscopy.

Anneal Time (min)	A_1	τ_1 (ns)	A_2	τ_2 (ns)	τ_{eff} (ns)
0	1.00	85	0.28	27873	6164
5	1.38	113	0.23	69046	9961
10	1.74	103	0.30	26634	4005
15	1.12	122	0.25	38611	7146
20	1.55	210	0.17	57204	5843
30	0.90	103	0.29	26189	6460
40	0.78	98	0.23	33747	7761
50	1.57	77	0.32	40360	6897
60	0.64	97	0.30	35881	11517

Supplementary Note 5. Defect Calculations

To rule out the possibility of the high prevalence of localised trap states in $NaBiS_2$ being the result of high concentrations of Na vacancies V_{Na}, defect formation energies were calculated for these species in both the ordered ($R\bar{3}m$) and disordered ($Fm\bar{3}m$) polymorphs. Formation energies were calculated using the conventional supercell approach, as detailed in Ref. 19–21.

For the ordered ($R\bar{3}m$) polymorph, a 56-atom supercell (13.7×10.7×10.7 Å^3) was used, generated using the optimal cubic supercell algorithm implemented in ASE[22,23], which gave a transformation matrix:

$$T = \begin{bmatrix} 1 & 1 & 0 \\ -3 & 2 & 1 \\ -2 & -1 & 3 \end{bmatrix}$$

for the primitive $R\bar{3}m$ cell. For the disordered ($Fm\bar{3}m$) system, the 80-atom SQS supercell described previously was used, and Na vacancies were created at each of the 20 Na sites in the supercell, to model the range of potential coordination environments in the disordered material. These 20 potential vacancy configurations were initially relaxed using Γ-point only reciprocal space sampling, from which 10 of the highest and lowest energy configurations were then further relaxed with the fully-converged k-point mesh before calculating the total energies using HSE06+SOC in both the neutral and -1 charge states (V_{Na}^{0} and V_{Na}^{-1}). This approach was validated by comparing the formation energies of the vacancies calculated with Γ-point only and fully-converged k-point sampling, for which the relative energies differed by less than 0.05 eV (Supplementary Fig. 22). The ShakeNBreak distortion sampling approach was additionally tested for these defects, to ensure the ground-state structures of the defects were correctly identified[24,25].

The same well-converged plane-wave energy cutoff (350 eV) and reciprocal space sampling was employed as for the earlier calculations. Each defective supercell was relaxed to the same ionic force convergence criteria (0.01 eV Å^{-1}) as for bulk structure optimisation, allowing spin-polarisation, prior to a static total-energy calculation including spin-orbit coupling effects (HSE06+SOC). To account for spurious finite-size supercell effects, the Freysoldt image charge and potential alignment correction scheme was implemented.

The computed phase stability map for $NaBiS_2$ is shown in Supplementary Fig. 19, which was used to determine the Na-rich and Na-poor chemical potential limits. Here the ordered ($R\bar{3}m$) phase of $NaBiS_2$ is used, due to the temperature-dependent entropy contribution on the formation energy of disordered $NaBiS_2$, though the small calculated order/disorder energy difference of 22 meV atom^{-1} means that using the athermal formation energy of disordered $NaBiS_2$ gives a negligible change in the chemical potential limits.

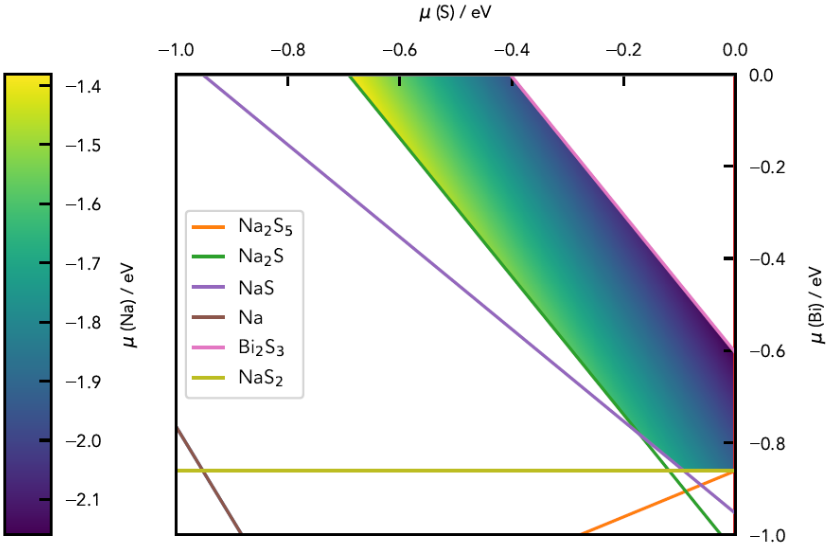

Supplementary Figure 19. Computed phase stability map of $NaBiS_2$ with HSE06+SOC, used to determine the Na-poor and Na-rich chemical potential limits for

defect calculations. The chemical potential (μ) of Na is set as the dependent variable, shown as the colour bar.

In ordered ($R\bar{3}m$) $NaBiS_2$, Na vacancies are found to be shallow acceptor defects as expected, with a (0/-1) transition level just above the VBM and likely contributing to *p*-type behaviour in this material. As shown in Supplementary Fig. 20, the formation energies of Na vacancies in ordered ($R\bar{3}m$) $NaBiS_2$ vary between 1.2 and 2.0 eV in the neutral charge state, depending on synthesis conditions (Na-poor *vs*. Na-rich).

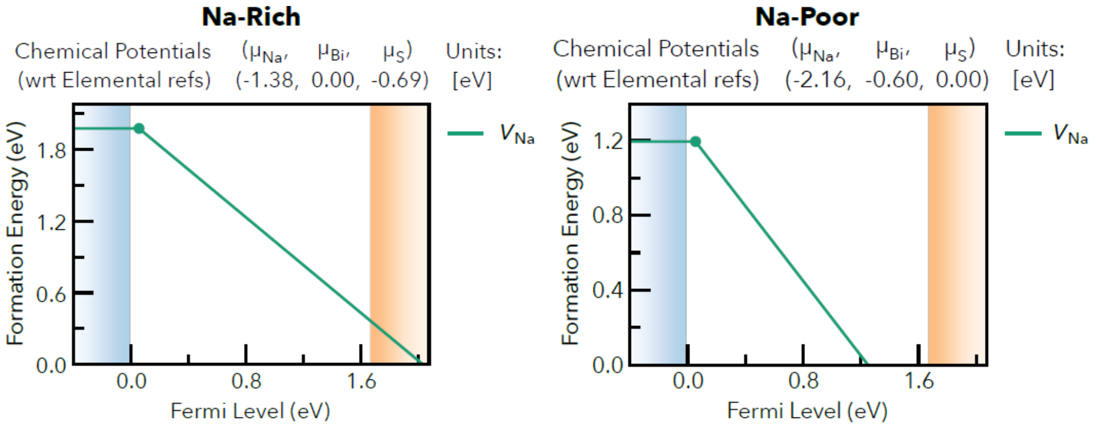

Supplementary Figure 20. Defect formation energy diagram for Na vacancies in ordered $R\bar{3}m$ $NaBiS_2$ calculated using HSE06+SOC, under both Na-rich and Na-poor chemical conditions. Valence band in blue, conduction band in orange.

In disordered ($Fm\bar{3}m$) $NaBiS_2$, the energy of the Na vacancy additionally depends on the local environment of the vacancy site, which differs due to the random cation distribution. Supplementary Fig. 21 shows the variation in V_{Na} formation energy, under Na-poor conditions, for different Na sites in the 80-atom SQS supercell as a function of distance from the localised S *p* state.

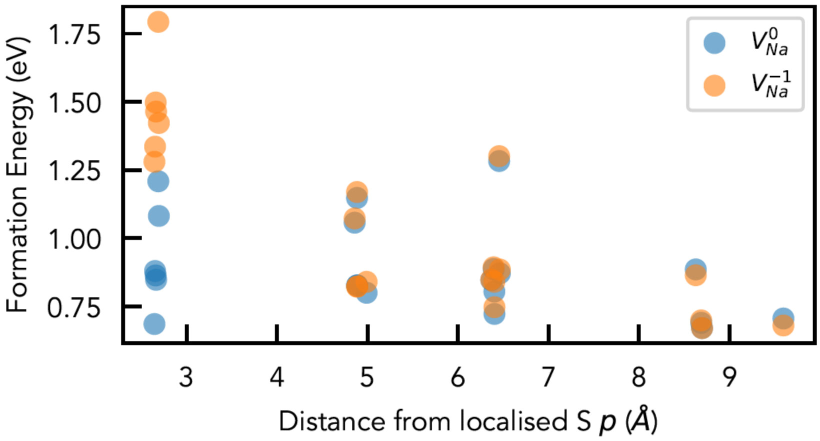

Supplementary Figure 21. Formation energies of neutral ($V_{\mathrm{Na}}{}^{0}$) and negatively-charged ($V_{\mathrm{Na}}{}^{-1}$) Na vacancies in disordered (SQS) $Fm\bar{3}m$ $NaBiS_2$, as a function of vacancy site distance from the localised S *p* state (see Fig. 2 in main text), using Γ-only k-point sampling and Na-poor chemical conditions. Formation energies for the negatively-charged vacancies ($V_{\mathrm{Na}}{}^{-1}$) correspond to a Fermi level positioned at the (0/-1) charge transition level of the lowest energy vacancy (i.e. where $E_{\mathrm{F}}(V_{\mathrm{Na}}{}^{0}) = E_{\mathrm{F}}(V_{\mathrm{Na}}{}^{-1})$).

The V_{Na} formation energies additionally calculated with fully-converged k-point sampling and spin-orbit coupling are shown in Supplementary Fig. 22.

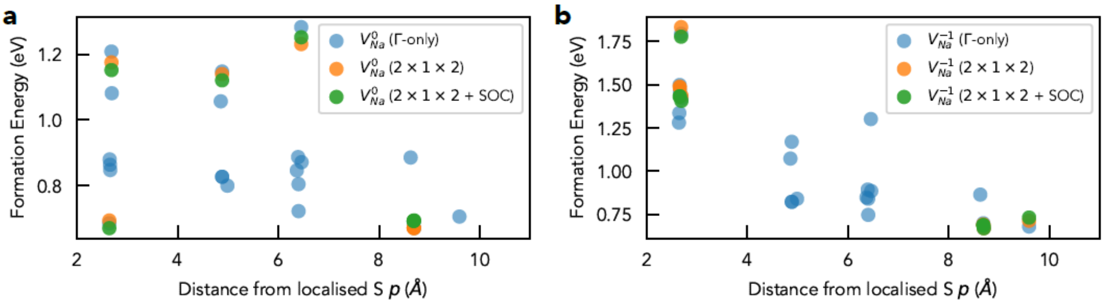

Supplementary Figure 22. Formation energies of **a** $V_{\mathrm{Na}}{}^{0}$ and **b** $V_{\mathrm{Na}}{}^{-1}$ in disordered (SQS) $Fm\bar{3}m$ $NaBiS_2$ as a function of vacancy site distance from the localised S *p* state (see Fig. 2a, main text), using Γ-only and fully-converged (2×1×2) k-point sampling, with and without spin-orbit coupling (SOC). Na-poor chemical conditions assumed.

Formation energies for the negatively-charged vacancies (V_{Na}^{-1}) correspond to a Fermi level positioned at the (0/-1) charge transition level of the lowest energy vacancy (*i.e.*, where the Fermi level $E_F(V_{Na}^0) = E_F(V_{Na}^{-1})$).

As shown in Supplementary Fig. 23, as expected, the Na vacancy formation energies in disordered $NaBiS_2$ are mostly lower than those in the ordered ($R\bar{3}m$) polymorph, now ranging from 0.7 to 1.2 eV, compared to 1.2 eV under the same Na-poor chemical conditions for the ordered structure (Supplementary Fig. 20).

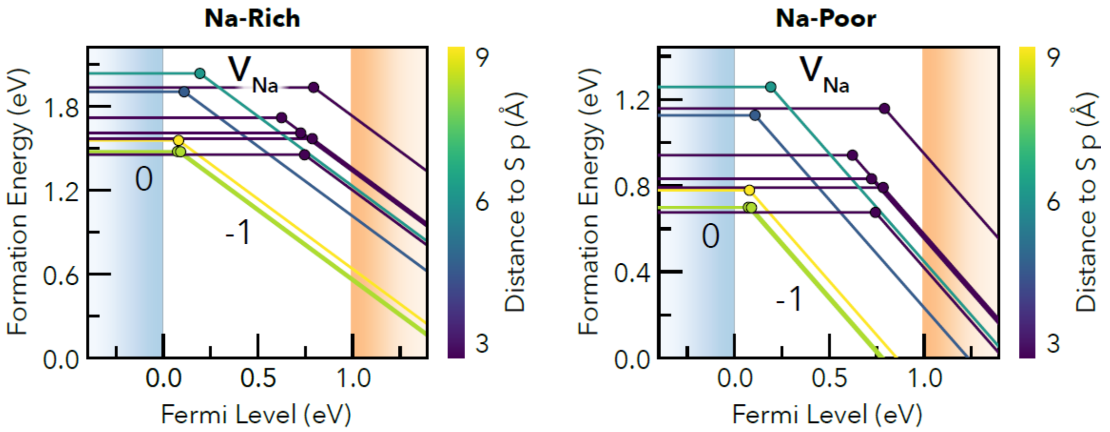

Supplementary Figure 23. Defect formation energy diagram for Na vacancies in disordered (SQS) $Fm\bar{3}m$ $NaBiS_2$, calculated using HSE06+SOC, under both Na-rich and Na-poor chemical conditions. Formation energy lines are colour-mapped to the vacancy site distance from the localised S *p* state (see Fig. 2 in main text). Valence band in blue, conduction band in orange.

The relative energies for V_{Na}^0 and V_{Na}^{-1}, for vacancy sites located away from the localised S *p* state, stay consistent (Supplementary Fig. 21 and 22), thus giving closely-spaced (0/-1) charge transition levels in the region 0.07–0.20 eV above the VBM. For the 6 Na sites surrounding the localised S *p* state (at ~2.5Å in Supplementary Fig. 21 and 22), however, we see a wider range of formation energies (spanning over ~0.5 eV),

depending on which Na site the vacancy is placed. Moreover, we also see that the -1 charge vacancies (V_{Na}^{-1}) are strongly disfavoured here, thus yielding much deeper (0/-1) charge transition levels in the region of 0.6–0.8 eV above the VBM. This behaviour can be chemically rationalised by considering the reason for the high energy of the localised S *p* state (Supplementary Fig. 5), having no neighbouring covalent bonding and reduced Coulomb attraction to the surrounding lower valence cations (Na^{+} rather than Bi^{3+}). Now, placing a negatively-charged vacancy next to this site further increases the electronic potential at this site, significantly increasing the energy and thus disfavouring V_{Na}^{-1} over V_{Na}^{0}. This behaviour is confirmed by the density of states (DOS) plots in Supplementary Fig. 24, which shows higher energy occupied in-gap S *p* states for V_{Na}^{-1} when located next to the localised S *p* state (with a distance $d \sim 2.5$ Å), but lower in energy (just above the VBM) when V_{Na}^{-1} is further away ($d \geq 2.5$ Å).

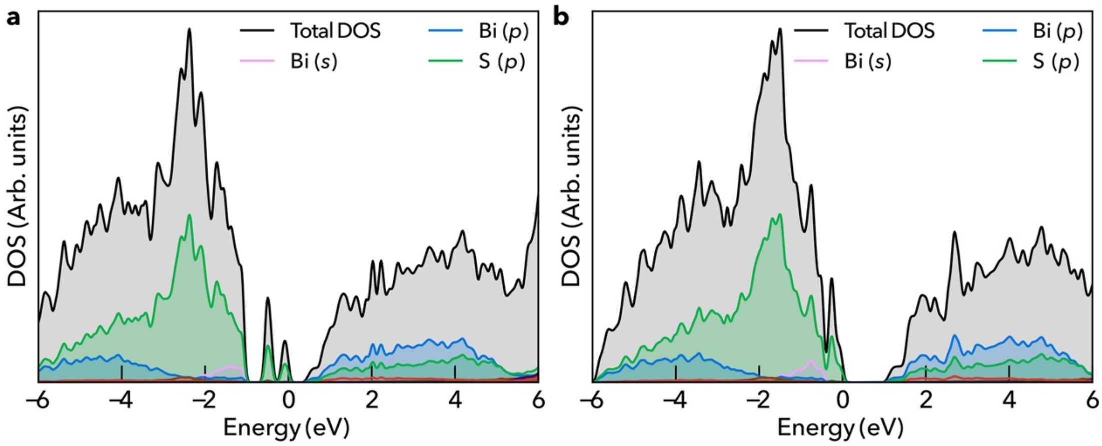

Supplementary Figure 24. Electronic density of states (DOS) for V_{Na}^{-1} in disordered (SQS) $Fm\bar{3}m$ $NaBiS_2$, calculated using HSE06+SOC, for V_{Na}^{-1} located next to the localised S *p* state **a** ($d \sim 2.5$ Å), and farther away **b** ($d \geq 2.5$ Å). Energy of the highest occupied state set to 0 eV.

For V_{Na}^0 however, this in-gap state is unoccupied and so there is much less energy difference between the vacancies located near or far from the S *p* state. Thus for V_{Na} located near S *p*, the relative destabilisation of the negative state results in a (0/-) transition level much higher in the bandgap.

Supplementary References

1. Gabrel'yan, B.V., Lavrentiev, A. A., Nikiforov, I. Y. & Sobolev, V.V. Electronic energy structure of $MBiS_2$ (M = Li, Na, K) calculated with allowance for the difference between the M-S and Bi-S bond lengths. *J. Struct. Chem.* **49**, 788–794 (2008).
2. Wang, Y. *et al.* Cation disorder engineering yields $AgBiS_2$ nanocrystals with enhanced optical absorption for efficient ultrathin solar cells. *Nat. Photonics* **16**, 235–241 (2022).
3. Laitinen, M., Rossi, M., Julin, J. & Sajavaara, T. Time-of-flight - Energy spectrometer for elemental depth profiling - Jyväskylä design. *Nucl. Instrum. Methods Phys. Res. B NUCL INSTRUM METH B* **337**, 55–61 (2014).
4. Arstila, K. *et al.* Potku - New analysis software for heavy ion elastic recoil detection analysis. *Nucl. Instrum. Methods Phys. Res. B NUCL INSTRUM METH B* **331**, 34–41 (2014).
5. Łukaszewicz, K., Stękepień-Damm, J., Pietraszko, A., Kajokas, A. & Grigas, J. Crystal structure, thermal expansion, dielectric permittivity and phase transitions of Bi_2S_3. *Pol. J. Chem.* **73**, 541–546 (1999).
6. Peng, Y. *et al.* Lead-Free Perovskite-Inspired Absorbers for Indoor Photovoltaics. *Adv. Energy Mater.* **11**, 2002761 (2021).

7. Slavney, A. H., Hu, T., Lindenberg, A. M. & Karunadasa, H. I. A Bismuth-Halide Double Perovskite with Long Carrier Recombination Lifetime for Photovoltaic Applications. *J. Am. Chem. Soc.* **138**, 2138–2141 (2016).

8. Bartesaghi, D. *et al.* Charge Carrier Dynamics in $Cs_2AgBiBr_6$ Double Perovskite. *J. Phys. Chem. C* **122**, 4809–4816 (2018).

9. Rosales, B. A., White, M. A. & Vela, J. Solution-Grown Sodium Bismuth Dichalcogenides: Toward Earth-Abundant, Biocompatible Semiconductors. *J. Am. Chem. Soc.* **140**, 3736–3742 (2018).

10. Buizza, L. R. V. *et al.* Charge-Carrier Mobility and Localization in Semiconducting Cu_2AgBiI_6 for Photovoltaic Applications. *ACS Energy Lett.* **6**, 1729–1739 (2021).

11. Wright, A. D. *et al.* Ultrafast Excited-State Localization in $Cs_2AgBiBr_6$ Double Perovskite. *J. Phys. Chem. Lett.* **12**, 3352–3360 (2021).

12. Elliott, R. J. Intensity of optical absorption by excitons. *Phys. Rev.* **108**, 1384–1389 (1957).

13. Saba, M. *et al.* Correlated electron-hole plasma in organometal perovskites. *Nat. Commun.* **5**, 5049 (2014).

14. Davies, C. L. *et al.* Bimolecular recombination in methylammonium lead triiodide perovskite is an inverse absorption process. *Nat. Commun.* **9**, 293 (2018).

15. Baqais, A., Tymińska, N., LeBahers, T. & Takanabe, K. Optoelectronic Structure and Photocatalytic Applications of $Na(Bi,La)S_2$ Solid Solutions with Tunable Band Gaps. *Chem. Mater.* **31**, 3211–3220 (2019).

16. Yang, Z. *et al.* Ultrafast self-trapping of photoexcited carriers sets the upper limit on antimony trisulfide photovoltaic devices. *Nat. Commun.* **10**, 4540 (2019).

17. Wehrenfennig, C., Eperon, G. E., Johnston, M. B., Snaith, H. J. & Herz, L. M. High charge carrier mobilities and lifetimes in organolead trihalide perovskites. *Adv. Mater.* **26**, 1584–1589 (2014).

18. Joyce, H. J., Boland, J. L., Davies, C. L., Baig, S. A. & Johnston, M. B. A review of the electrical properties of semiconductor nanowires: Insights gained from terahertz conductivity spectroscopy. *Semicond. Sci. Technol.* **31**, 103003 (2016).

19. Freysoldt, C. *et al.* First-principles calculations for point defects in solids. *Rev. Mod. Phys.* **86**, 253–305 (2014).

20. Kavanagh, S. R., Walsh, A. & Scanlon, D. O. Rapid Recombination by Cadmium Vacancies in CdTe. *ACS Energy Lett.* **6**, 1392–1398 (2021).

21. Huang, Y. T., Kavanagh, S. R., Scanlon, D. O., Walsh, A. & Hoye, R. L. Z. Perovskite-inspired materials for photovoltaics and beyond-from design to devices. *Nanotechnology* **32**, 132004 (2021).

22. Erhart, P., Sadigh, B., Schleife, A. & Åberg, D. First-principles study of codoping in lanthanum bromide. *Phys. Rev. B* **91**, 165206 (2015).

23. Hjorth Larsen, A. *et al.* The atomic simulation environment - A Python library for working with atoms. *J. Phys. Condens. Matter* **29**, 273002 (2017).

24. Mosquera-Lois, I. & Kavanagh, S. R. In search of hidden defects. *Matter* **4**, 2602–2605 (2021).

25. Mosquera-Lois, I., Kavanagh, S. R., Walsh, A., Scanlon, D. O. Identifying the ground state structures of point defects in solids. arXiv: 2207.09862v2 https://doi.org/10.48550/arXiv.2207.09862 (2022).
